# Health-Related Social Needs Among LGB+ Veterans

**DOI:** 10.1001/jamanetworkopen.2025.39986

**Published:** 2025-10-29

**Authors:** Shane Lamba, David A. Frank, Jennifer L. McCoy, Soumik Purkayastha, Sarah M. Leder, Lauren E. Russell, Joshua H. Gordon, Gregory T. Procario, Ernest M. Moy, Leslie R. M. Hausmann

**Affiliations:** 1 Office of Health Equity, Veterans Health Administration, Washington, DC; 2 Center for Healthcare Evaluation, Research, and Promotion (CHERP), Veterans Affairs Pittsburgh Healthcare System, Pittsburgh, Pennsylvania; 3 Department of Biostatistics and Health Data Science, University of Pittsburgh School of Public Health, Pittsburgh, Pennsylvania; 4 Mental Illness Research, Education, and Clinical Center (MIRECC), Veterans Affairs Pittsburgh Healthcare System, Pittsburgh, Pennsylvania; 5 Department of Medicine, University of Pittsburgh School of Medicine, Pittsburgh, Pennsylvania

## Abstract

**Importance:**

Veterans identifying as lesbian, gay, bisexual, additional orientations, or not sure (LGB+) may have unique health-related social needs. By addressing social needs, health systems can take steps to alleviate persistent health disparities in this population.

**Objective:**

To evaluate associations between sexual orientation and need for support for social needs among primary care patients served by the Veterans Health Administration (VHA).

**Design, Setting, and Participants:**

This cross-sectional study used data from a national sample of VHA primary care patients seen in January or February 2023 who were invited to participate in a survey online or by mail. Data collection occurred from March 2 through May 9, 2023.

**Exposure:**

Self-reported sexual orientation (straight or LGB+).

**Main Outcomes and Measures:**

Unadjusted prevalence ratios (PRs) and adjusted PRs (APRs) of need for support across 13 health-related social domains.

**Results:**

Of 38 759 veterans invited to participate in the survey, 7095 (18.3%) responded and 6296 (16.2%) with complete data were included. Weighted responses represented 903 714 veterans. The unweighted number (weighted percentage) identifying as straight was 5874 (94.2%) and as LGB+ was 422 (5.8%); 3585 (89.1%) were male. In unadjusted comparisons, LGB+ veterans had a higher prevalence of need for support for feeling socially isolated (PR, 1.40; 95% CI, 1.04-1.87), feeling lonely (PR, 1.59; 95% CI, 1.20-2.09), finding or keeping work (PR, 1.71; 95% CI, 1.07-2.75), paying for food (PR, 1.71; 95% CI, 1.19-2.46), paying for basics such as housing, medical care, and heating (PR, 1.89; 95% CI, 1.29-2.75), managing experiences of discrimination (PR, 2.31; 95% CI, 1.54-3.48), and getting or maintaining housing (PR, 2.41; 95% CI, 1.40-4.15). After adjusting for age and for a combined race, ethnicity, and sex variable, LGB+ veterans had higher prevalence of need for support for managing experiences of discrimination (APR, 1.79; 95% CI, 1.21-2.64) and getting or maintaining housing (APR, 1.91; 95% CI, 1.10-3.34).

**Conclusions and Relevance:**

This cross-sectional study found that LGB+ veterans reported a higher prevalence of social needs related to managing experiences of discrimination and housing instability compared with their straight counterparts. Expansion of support systems to address social needs in general and to ensure that systems are tailored for groups that more often experience certain needs should be considered.

## Key Points

**Question:**

Does the prevalence of 13 health-related social needs differ by sexual orientation among US veterans?

**Findings:**

In this cross-sectional study of a national sample of 6296 primary care patients served by the Veterans Health Administration, veterans who identified as lesbian, gay, bisexual, additional orientations, or not sure (LGB+) endorsed a greater need for support for managing experiences of discrimination and getting or maintaining housing compared with those who identified as straight.

**Meaning:**

While most health-related social needs did not differ by sexual orientation, the findings suggest LGB+ veterans have need for increased support around discriminatory experiences and housing.

## Teaser

This cross-sectional study investigates associations between sexual orientation and need for support for social needs among patients served by the Veterans Health Administration.

## Introduction

It has been nearly 15 years since the repeal of Don’t Ask, Don’t Tell, a policy that allowed nonstraight individuals to remain in the US military as long as they did not reveal their sexual orientation.^1,2^ During that time, the Veterans Health Administration (VHA) has taken steps to expand services, improve patient experiences, and reduce health disparities for veterans identifying as lesbian, gay, bisexual, additional nonstraight sexual orientations, or not sure (LGB+).^3,4,5,6^ Recent studies have identified that LGB+ veterans continue to have worse health outcomes^7,8^ and have less-positive health care experiences within VHA compared with straight veterans.^9,10^ A small but growing body of literature has identified differences in social factors that may be important drivers of observed health disparities. For example, studies investigating social risk factors, defined as specific, individual-level adverse social conditions associated with poor health,^11^ have identified a greater risk of food insecurity, housing instability, discrimination, and financial distress among LGB+ individuals.^12,13,14,15,16^ To our knowledge, health-related social needs (hereafter, *social needs*), which incorporate one’s perceptions of need and priorities for seeking or accepting support,^11^ have not yet been compared between LGB+ veterans and their straight counterparts. Understanding differences in self-reported need for support, as opposed to being at risk for experiencing a need, is an important step toward creating and tailoring support systems. Calls for advancing research in the field of social care equity have emphasized the importance of considering social needs in the context of marginalized identities in order to develop effective and appropriate social care interventions.^17^

With its national reach, diverse resources and programs, and relationships with community partners, the VHA is uniquely positioned to address the social needs of LGB+ veterans. This population is estimated to comprise approximately 2% to 4% of the larger veteran population.^9,18,19^ LGB+ sexual orientations are more prevalent among younger veterans, women veterans, and veterans from minoritized racial or ethnic backgrounds.^9,18,20^ Recent VHA initiatives have been implemented to improve services for LGB+ veterans, such as the creation of Lesbian, Gay, Bisexual, Transgender, Queer, and Additional Identities (LGBTQ+) Veteran Care Coordinators,^5^ the LGBTQ+ Health Program,^21^ and directives to provide affirming care.^6,22^ With the expansion of social screening tools, including Assessing Circumstances and Offering Resources for Needs (ACORN),^23^ the VHA can make greater strides in improving care for LGB+ veterans by assessing their unique social needs.

The purpose of this study was to investigate how self-reported need for support in 13 social need domains varied by sexual orientation in veterans receiving VHA primary care services. This study adds to the literature on veterans’ social needs by estimating the prevalence of self-reported need for support across a wide range of social domains among LGB+ individuals in a national patient population. Our findings can inform the development of social care programs aimed at marginalized populations.

## Methods

This cross-sectional study was deemed exempt from human participants research oversight and collection of informed consent by the institutional review board at the VA Pittsburgh Healthcare System because we used deidentified data collected for nonresearch purposes through the Department of Veterans Affairs (VA) Survey of Healthcare Experiences of Patients program. We followed the Strengthening the Reporting of Observational Studies in Epidemiology (STROBE) reporting guideline.^24^

### Study Design and Survey Administration

This was a secondary cross-sectional analysis of data collected online or by mail via the VA’s Your Recent Visit survey, an instrument to assess patient experiences as part of the VA’s Survey of Healthcare Experiences of Patients program. The parent study was designed to examine race, ethnicity, and sex differences in self-reported social risks and needs among VHA primary care patients.^25^ The sampling frame for the parent study was constructed to yield approximately 900 respondents from 6 mutually exclusive racial, ethnic, and sex groups consisting of Black, Hispanic, and White males and females who had VHA primary care encounters in January or February 2023. More details on the survey administration have been published previously.^25^ The current analysis used data from respondents who completed the social needs section of the survey and provided a sexual orientation. Data collection occurred from March 2 through May 9, 2023.

### Primary Exposure—Sexual Orientation

Respondents were asked to report their sexual orientation by choosing 1 of the following: lesbian, gay, straight, bisexual, other (hereafter, *additional orientations*), or not sure. We combined responses of lesbian, gay, bisexual, additional orientations, or not sure into a single category (LGB+). Previous research has indicated that individuals who are unsure of their sexual orientation have similar response patterns as LGB+ individuals to social risk questionnaires.^26,27^ Respondents reporting their sexual orientation as straight served as the reference group. Those who did not respond to the question were excluded.

### Outcomes—Need for Support

Participants were asked, “In the past 6 months, did you need support with any of the following?” Choices were “paying for basics such as food, housing, medical care, and heating,” “obtaining adult caregiving for yourself or others,” “obtaining childcare,” “finding or keeping work,” “paying for food,” “getting or maintaining housing,” “getting transportation for basic needs like medical care or grocery shopping,” “accessing internet at home,” “feeling socially isolated,” “feeling lonely,” “managing experiences of discrimination,” “getting assistance with legal issues,” and “getting additional education or job training.” For each domain, participants were provided 3 response options: “no support needed,” “needed support and got it,” and “needed support but did not get it.” For analyses, the latter 2 responses were combined into a single category of “needed support.”

### Covariates

Previous study samples have shown that younger veterans are more likely to identify as LGB+ compared with older veterans, which led us to include age group as a covariate in adjusted models.^9,20^ Self-reported age category (18-44, 45-54, 55-64, 65-74, or ≥75 years) was supplemented using sampling data for respondents with item nonresponse. Those aged 18 to 44 years were combined into 1 category due to few respondents in the youngest categories.

We also included a combined self-reported race, ethnicity, and sex variable as a covariate in adjusted models due to our group’s prior findings of significant differences in the prevalence of social needs by race, ethnicity, and sex in this sample.^25^ Respondents were asked to select 1 or more races from options including American Indian or Alaska Native, Asian, Black or African American, Native Hawaiian or Other Pacific Islander, and White. Due to small numbers, we excluded respondents who were categorized as non-Hispanic American Indian or Alaska Native, Asian, or Native Hawaiian or Other Pacific Islander or who selected multiple races. For ethnicity, respondents were asked if they were of Hispanic or Latino origin or descent (yes or no). Respondents who self-reported as Hispanic or Latino were categorized as Hispanic regardless of their response to the race question. For brevity, non-Hispanic Black and non-Hispanic White will be referred to as *Black* and *White*, respectively, hereafter. Sex was assessed by the question, “What sex is listed on your birth certificate?” Answer choices were female or male. Respondents with missing responses for race, ethnicity, and sex were assigned to groups using administrative data.

### Statistical Analysis

Survey weights were applied to account for the sampling frame and survey nonresponse. Weighted data were used for all analyses, and weighted percentages are reported. We used Rao-Scott second-order corrected Pearson χ^2^ tests to test for differences in demographics across groups. Unadjusted prevalence of need for support for each domain was reported for the overall sample and stratified by sexual orientation.

We developed log-binomial models to examine the association between sexual orientation and need for support in each domain. For all models, we performed unadjusted analyses and analyses adjusted for age category and for the combined race, ethnicity, and sex variable, using straight veterans as the reference group. For the covariates, the largest categories were used as the reference categories (ie, age of 65-74 years and White males). Prevalence ratios (PRs) and adjusted PRs (APRs) were calculated from the models to avoid overestimating effect sizes in a cross-sectional study.^28^ Two-sided *P* values less than .05 were considered statistically significant. Data extraction, cleaning, analyses, and figure creation were performed using R, version 4.3.2 (R Project for Statistical Computing).

## Results

### Sample Characteristics

**Figure 1.**
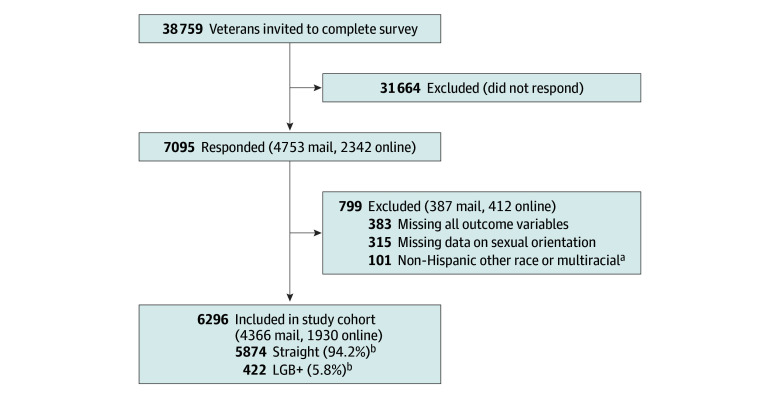
Flowchart for Analytic Cohort LGB+ indicates lesbian, gay, bisexual, additional orientations, or not sure. ^a^Non-Hispanic other race includes veterans who were categorized as American Indian or Alaska Native, Asian, or Native Hawaiian or Other Pacific Islander. ^b^Data are expressed as unweighted number of participants and weighted percentages.

A total of 38 759 veterans were invited to participate and 7095 (18.3%) responded. Respondents were more likely to be White, male, and aged 55 years or older than were nonrespondents (eTable 1 in Supplement 1). After excluding respondents with missing data on all outcomes and sexual orientation, the final sample included 6296 veterans (16.2% of those invited) (Figure 1).

The weighted sample of 6296 respondents represented 903 714 veterans. The unweighted number (weighted percentage) who identified as straight was 5874 (94.2%) and as LGB+ was 422 (5.8%); there were 2711 (10.9%) females and 3585 (89.1%) males. By the combined race, ethnicity, and sex variable, the unweighted numbers and weighted percentages of respondents were 1040 Black females (4.0%), 1083 Black males (19.3%), 894 Hispanic females (1.6%), 1206 Hispanic males (11.2%), 777 White females (5.3%), and 1296 White males (58.6%). Respondents with missing responses for age (15 [1.2%]), race (356 [2.9%]), ethnicity (128 [3.5%]), and sex (9 [1.4%]) were assigned to groups using administrative data. Compared with straight veterans, veterans that identified as LGB+ tended to be younger and were more likely to be Black females, Hispanic females, Hispanic males, or White females (Table).

**Table.** Sociodemographic Characteristics of Survey Respondents Overall and by Sexual Orientation

Characteristic	Respondents^a^			*P* value
	Overall	Straight	LGB+	
Participants, No.				
Unweighted	6296	5874	422	NA
Weighted	903 714	851 405	52 309	NA
Age, y^b^				
18-44	573 (16.7)	477 (15.3)	96 (39.0)	<.001
45-54	764 (12.7)	709 (12.6)	55 (13.6)	
55-64	1546 (19.3)	1421 (19.4)	125 (17.5)	
65-74	1908 (25.4)	1817 (26.1)	91 (14.5)	
≥75	1505 (25.9)	1450 (26.6)	55 (15.4)	
Race, ethnicity, and sex^b^^,^^c^				
Black female	1040 (4.0)	963 (3.8)	77 (7.5)	<.001
Black male	1083 (19.3)	1048 (19.6)	35 (15.0)	
Hispanic female	894 (1.6)	786 (1.3)	108 (6.9)	
Hispanic male	1206 (11.2)	1158 (10.9)	48 (17.1)	
White female	777 (5.3)	673 (4.6)	104 (17.4)	
White male	1296 (58.6)	1246 (60.0)	50 (36.1)	

Abbreviations: LGB+, lesbian, gay, bisexual, additional orientations, or not sure; NA, not applicable.

^a^ Data are expressed as unweighted number of participants (weighted percentages) unless otherwise indicated.

^b^ Missing self-reported data were supplemented with administrative sampling data (included 15 participants [1.2%] missing age data, 356 [2.9%] missing race data, 128 [3.5%] missing ethnicity data, and 9 [1.4%] missing sex data).

^c^ Included as a combined variable. Sex was assessed by the question, “What sex is listed on your birth certificate?” Answer choices were female or male. All respondents who reported as Hispanic or Latino were categorized as Hispanic regardless of race.

### Prevalence of Need for Support Among LGB+ Veterans

**Figure 2.**
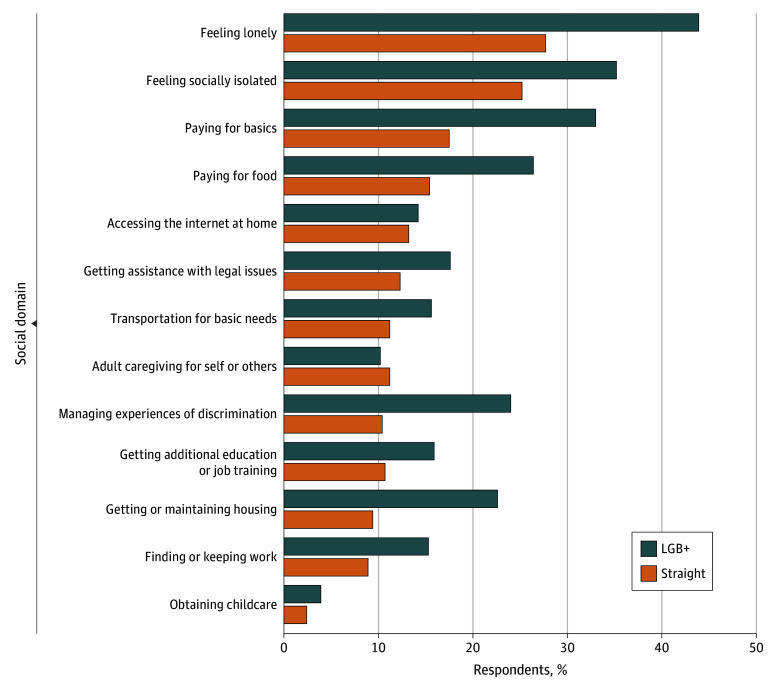
Unadjusted Prevalence of Need for Support for Social Domains, Stratified by Self-Reported Sexual Orientation Need for support was defined as responding “needed support and got it” or “needed support but did not get it.” Data were self-reported and expressed as weighted percentages. Straight: unweighted n = 5874, weighted n = 851 405. Lesbian, gay, bisexual, additional orientations, or not sure (LGB+): unweighted n = 422, weighted n = 52 309.

**Figure 3.**
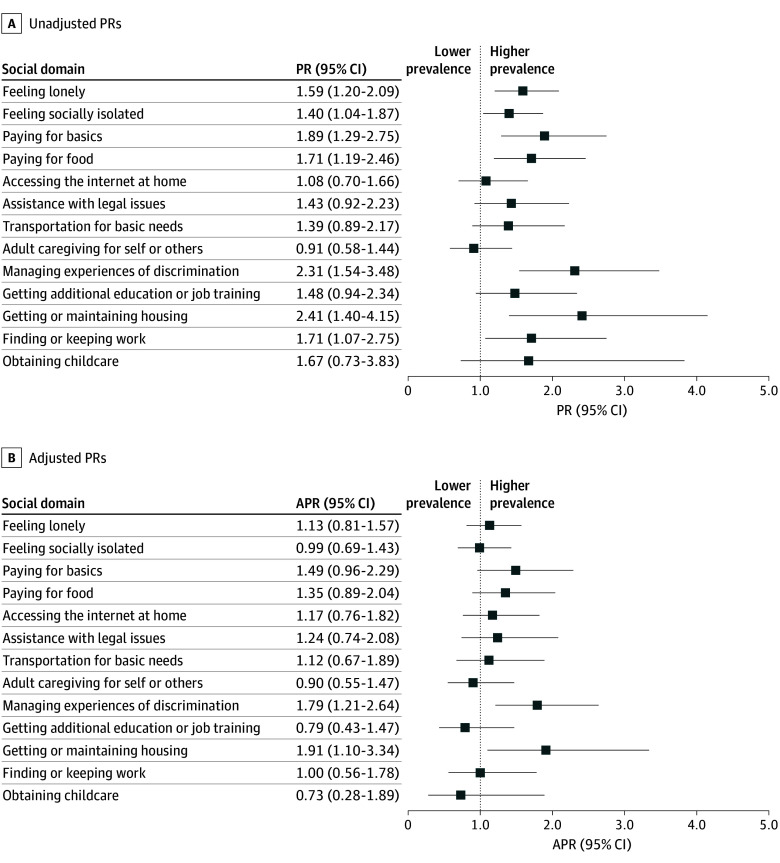
Unadjusted and Adjusted Prevalence Ratios (PRs) of Need for Support in Social Domains for LGB+ Compared With Straight Veterans Adjusted PRs (APRs) were adjusted for age group, race, ethnicity, and sex. LGB+ indicates lesbian, gay, bisexual, additional orientations, or not sure.

Prior to adjustment for age or for race, ethnicity, and sex, need for support ranged from weighted percentages of 2.5% for obtaining childcare to 28.6% for feeling lonely in the overall sample (Figure 2) and were higher among LGB+ veterans for all domains except adult caregiving (eg, 43.9% of LGB+ veterans reported needing support for feeling lonely compared with 27.7% of straight veterans) (Figure 2). Additionally, LGB+ veterans were over twice as likely to report needing support compared with straight veterans for managing experiences of discrimination (24.0% vs 10.4%) and getting or maintaining housing (22.6% vs 9.4%) (Figure 2). In unadjusted models, there were significant differences in need for support between LGB+ and straight veterans in 7 domains (Figure 3 and eTable 2 in Supplement 1). Specifically, need for support was significantly more prevalent among LGB+ (vs straight) veterans for feeling lonely (PR, 1.59; 95% CI, 1.20-2.09), social isolation (PR, 1.40; 95% CI, 1.04-1.87), paying for basics (PR, 1.89; 95% CI, 1.29-2.75), paying for food (PR, 1.71; 95% CI, 1.19-2.46), managing experiences of discrimination (PR, 2.31; 95% CI, 1.54-3.48), getting or maintaining housing (PR, 2.41; 95% CI, 1.40-4.15), and finding or keeping work (PR, 1.71; 95% CI, 1.07-2.75).

After adjustment for age and for race, ethnicity, and sex, significant differences remained in 2 domains (Figure 3 and eTable 2 in Supplement 1). Need for support remained more prevalent among LGB+ veterans in the domains of managing experiences of discrimination (APR, 1.79; 95% CI, 1.21-2.64) and getting or maintaining housing (APR, 1.91; 95% CI, 1.10-3.34).

## Discussion

This study contributes to the growing body of knowledge on the social needs of LGB+ veterans who receive care within the VHA. Our findings demonstrated that after adjusting for age and for race, ethnicity, and sex, LGB+ veterans had a higher prevalence of self-reported need for support in the domains of managing experiences of discrimination and getting or maintaining housing. For the remaining 11 social needs included in this study, need for support did not differ by sexual orientation after adjusting for other demographic differences.

Our findings build on previous research that identified discrimination as a persistent experience for LGB+ veterans receiving VHA services.^10,29,30,31,32,33^ The current study found that nearly 1 in 4 LGB+ veterans reported needing support for discrimination, compared with 1 in 10 straight veterans. Our findings are consistent with existing literature showing that LGB+ people, including veterans, experience heightened discrimination in their everyday life compared with straight peers.^10,29,30,31,32,33^ It is important to note that this study assessed need for support managing experiences of discrimination without specifying the setting in which discrimination occurred or reason for the discrimination. Other research has documented that discrimination affecting LGB+ communities often arises from homophobia and structural discrimination, which are associated with heightened chronic stress and well-documented health disparities.^7,34^ In a qualitative analysis, LGB+ veterans noted that common sources of discrimination and negative experiences include obtaining VA and non-VA health care, military culture, and interactions with members of other sexual minority groups.^10^ Furthermore, a recent analysis using data from the VA’s Survey of Healthcare Experiences of Patients found that LGB+ veterans were less likely than straight veterans to indicate having a positive experience with their VHA clinicians and more likely to report feeling that they were not listened to, were not respected, and were rushed through the appointment without the opportunity to discuss their health goals with their clinicians.^9^ Taken together, discrimination experienced by LGB+ veterans can be multifaceted, and any interventions to address the impact of discrimination should be carefully tailored to the needs of this population.^10^

We also found that the proportion of respondents needing support for finding or maintaining housing was higher among LGB+ veterans, which adds to the limited data available on the prevalence of needing support for housing instability within this population. For example, a recent analysis of screening for housing instability in VHA health care encounters found LGB+ veterans were nearly twice as likely to experience recent homelessness and housing instability compared with straight veterans.^26^ Our finding that more LGB+ veterans reported needing support for housing is consistent with research that has found the general LGB+ population to be more likely to struggle with housing instability.^12,13,35^ Furthermore, it is possible that there is overlap in experiences of discrimination and housing needs among LGB+ communities, which was not explored in this study but has been noted elsewhere.^36,37^ Future research should explore the intersectional relationships between discrimination, housing instability, and other social needs in the LGB+ community. The VHA has committed substantial resources to reducing veteran homelessness, such that homelessness among veterans has decreased at a much greater rate in the past decade than in the general population.^38^ Based on our findings, we recommend that steps be taken to ensure existing initiatives to combat housing instability among veterans are meeting the heightened need for support experienced by the LGB+ veteran community.

In addition, it is important to discuss the existing body of research on LGB+ social needs and the gaps filled by the present study. In our analysis of need for support across 13 social domains, LGB+ veterans had no significant differences compared with straight veterans in 11 domains, some of which have been previously identified as significantly impacting LGB+ individuals. For example, a recent analysis of over 3 million veterans receiving VHA services found that LGB+ veterans were more likely to experience food insecurity and housing instability.^26^ Need for support was not assessed in that study. Additionally, analyses using national surveys have found LGB+ individuals more likely to be at risk for food insecurity, housing instability, discrimination, financial distress, and social isolation.^12,13,14,15,16^ However, after we adjusted for age and for race, ethnicity, and sex, we did not identify significant need for support in the domains of food, paying for basics, and social isolation, as other studies have found.^12,13,14,15,16^ The findings in our analysis are not in conflict with and do not negate these previous analyses for an important reason: we asked participants to report on their need for support in each domain rather than asking if they had experienced or were worried that they may experience instability in a certain domain, a concept that is known as a social risk.^11^ Established research has identified a drop-off in the identification of social risks and the desire for assistance with those risks as well as the possibility for endorsed social needs to be in domains different from endorsed social risks.^39,40,41,42^ The purpose of the present study was to identify the prevalence of social needs in a population for which little research exists. Social risks and social needs are distinct concepts,^11^ and future social care interventions should incorporate an understanding of both concepts, especially in the context of marginalized communities.

### Strengths and Limitations

Our analysis has several strengths. We reported on need for support across a wide array of health-related social domains and focused on differences by sexual orientation. We oversampled women, who are more likely than men to identify as LGB+ in the veteran population,^9,19,20^ contributing to a greater representation of sexual minority individuals in our sample. LGB+ veterans represented 5.8% of our veteran sample, which is almost 2 times higher than found in a previous study using 2020 data from a similar VHA survey.^9^ Additionally, we controlled for demographic factors including age, race, ethnicity, and sex to account for potential confounding of associations of need for social support among veterans with LGB+ identities.

Our study also has limitations. Our sample included patients who used VHA primary care services and completed a postcare survey, limiting generalizability within the greater veteran population. The study’s reliance on self-reported data makes it susceptible to social desirability and recall biases. Individuals who did not report sexual orientation were excluded from the analysis because their data may not be missing at random and imputation would likely introduce bias.^43^ Additionally, we may have missed individuals who did not report sexual orientation on VHA surveys due to previous discriminatory policies (such as Don’t Ask, Don’t Tell) that may have created fear of disclosing sexual orientation.^44^ We acknowledge that combining LGB+ veterans into a single group may mask differences across subgroups; however, small sample sizes precluded examining variation in social needs at a more granular level. Due to the deidentified nature of the data, we were unable to control for other potential confounders. We also could not explore how or why LGB+ veterans in the study cohort needed support for discrimination and housing instability. Future qualitative or mixed-methods studies that gather more in-depth information could help inform future VHA programming.

## Conclusions

In this cross-sectional study of veterans, we found that LGB+ veterans reported experiencing a higher prevalence of social needs related to managing experiences of discrimination and housing instability compared with their straight counterparts. Our analysis underscores the need for more targeted and responsive systems to support LGB+ veterans experiencing these needs. The growing population of LGB+ veterans and the persistent social inequities they face call for enhanced interventions at both the national and local levels. Future research should continue to explore the multifaceted nature of discrimination, how to connect LGB+ veterans with support for housing needs, and how LGB+ subgroups differ in their social needs to develop effective social care interventions for this population.

## Notes

Supplement 1. **eTable 1.** Comparison of Survey Responders and Nonresponders **eTable 2.** Unadjusted and Adjusted Differences in Prevalence of Social Needs by Sexual Orientation

Supplement 2.
Data Sharing Statement
