# Supplementary material for: Health-Related Social Needs Among LGB+ Veterans
Source: JAMA Netw Open. 2025 Oct 29;8(10):e2539986. doi: 10.1001/jamanetworkopen.2025.39986 (PMC12573039; doi:10.1001/jamanetworkopen.2025.39986)
Supplement: Supplement 1. — eTable 1. Comparison of Survey Responders and Nonresponders eTable 2. Unadjusted and Adjusted Differences in Prevalence of Social Needs by Sexual Orientation [file jamanetwopen-e2539986-s001.pdf]

Supplementary Online Content

Lamba S, Frank DA, McCoy JL, et al. Health-related social needs among LGB+ veterans. *JAMA Netw Open*. 2025;8(10):e2539986. doi:10.1001/jamanetworkopen.2025.39986

eTable 1. Comparison of Survey Responders and Nonresponders

eTable 2. Unadjusted and Adjusted Differences in Prevalence of Social Needs by Sexual Orientation

This supplementary material has been provided by the authors to give readers additional information about their work.

© 2025 Lamba S et al. *JAMA Network Open*.

eTable 1: Comparison of Survey Responders and Nonresponders[a]

Characteristic	Survey Recipients		
	Non-Responder	Responder	P value
No. of participants	31,664	7,095	
Sampled strata			
Black Female	7801 (24.6)	1199 (16.9)	<0.001
Hispanic Female	7850 (24.8)	1059 (14.9)	
White Female	3549 (11.2)	851 (12.0)	
Black Male	4778 (15.1)	1222 (17.2)	
Hispanic Male	4660 (14.7)	1390 (19.6)	
White Male	3026 (9.6)	1374 (19.4)	
Age group			
18 - 34	4785 (15.1)	161 (2.3)	<0.001
35 - 44	7105 (22.4)	546 (7.7)	
45 - 54	6319 (20.0)	901 (12.7)	
55 - 64	6459 (20.4)	1736 (24.5)	
65 to 74	4669 (14.8)	2121 (29.9)	
75 or older	2327 (7.4)	1630 (23.0)	
Type of visit[b]			
By phone	5659 (17.9)	1171 (16.5)	<0.001
Video visit	2630 (8.3)	429 (6.1)	
In clinician's office	23365 (73.8)	5488 (77.4)	

[a] Data were extracted from the administrative record and are expressed as No.(%) unless otherwise indicated.
[b] Based on the visit type associated with the primary care visit between January and February 2023 connected with this survey.

© 2025 Lamba S et al. *JAMA Network Open*.

eTable 2: Unadjusted and Adjusted Differences in Prevalence of Social Needs by Sexual Orientation

	Unadjusted		Adjusted[a]	
	**Prevalence ratio [95% CI]**	**P-value**	**Prevalence ratio [95% CI]**	**P-value**
Feeling lonely				
Straight	1 [Reference]		1 [Reference]	
Lesbian, gay, bisexual, additional orientations, not sure	1.59 [1.20 to 2.09]	0.001	1.13 [0.81 to 1.57]	0.48
Black Female			1.58 [1.25 to 1.99]	<0.001
Hispanic Female			1.63 [1.22 to 2.17]	<0.001
White Female			1.23 [0.95 to 1.59]	0.11
Black Male			1.53 [1.23 to 1.90]	<0.001
Hispanic Male			1.44 [1.10 to 1.89]	0.009
White Male			1 [Reference]	
18 to 44			1.99 [1.54 to 2.57]	<0.001
45 to 54			1.83 [1.48 to 2.26]	<0.001
55 to 64			1.40 [1.16 to 1.68]	<0.001
65 to 74			1 [Reference]	
75 or older			0.71 [0.58 to 0.88]	0.002
Feeling socially isolated				
Straight	1 [Reference]		1 [Reference]	
Lesbian, gay, bisexual, additional orientations, not sure	1.40 [1.04 to 1.87]	0.02	0.99 [0.69 to 1.43]	0.96
Black Female			1.48 [1.17 to 1.88]	0.001
Hispanic Female			1.39 [1.03 to 1.88]	0.03
White Female			1.08 [0.83 to 1.42]	0.55
Black Male			1.35 [1.08 to 1.69]	0.009
Hispanic Male			1.22 [0.91 to 1.63]	0.19
White Male			1 [Reference]	
18 to 44			2.26 [1.71 to 3.00]	<0.001
45 to 54			2.13 [1.69 to 2.68]	<0.001
55 to 64			1.63 [1.33 to 2.01]	<0.001
65 to 74			1 [Reference]	
75 or older			0.62 [0.49 to 0.79]	<0.001
Paying for basics				
Straight	1 [Reference]		1 [Reference]	
Lesbian, gay, bisexual, additional orientations, not sure	1.89 [1.29 to 2.75]	<0.001	1.49 [0.96 to 2.29]	0.07
Black Female			1.42 [1.03 to 1.95]	0.03
Hispanic Female			1.23 [0.80 to 1.89]	0.34
White Female			0.96 [0.67 to 1.39]	0.83
Black Male			1.55 [1.18 to 2.04]	0.002
Hispanic Male			1.27 [0.87 to 1.85]	0.22
White Male			1 [Reference]	

© 2025 Lamba S et al. *JAMA Network Open*.

	Unadjusted		Adjusted[a]	
	**Prevalence ratio [95% CI]**	**P-value**	**Prevalence ratio [95% CI]**	**P-value**
Paying for basics (continued)				
18 to 44			1.55 [1.07 to 2.23]	0.02
45 to 54			1.22 [0.91 to 1.63]	0.19
55 to 64			1.03 [0.82 to 1.30]	0.81
65 to 74			1 [Reference]	
75 or older			0.50 [0.39 to 0.65]	<0.001
Paying for food				
Straight	1 [Reference]		1 [Reference]	
Lesbian, gay, bisexual, additional orientations, not sure	1.71 [1.19 to 2.46]	0.003	1.35 [0.89 to 2.04]	0.16
Black Female			1.62 [1.18 to 2.21]	0.003
Hispanic Female			1.40 [0.91 to 2.16]	0.12
White Female			1.17 [0.82 to 1.68]	0.39
Black Male			1.57 [1.18 to 2.08]	0.002
Hispanic Male			1.30 [0.90 to 1.88]	0.16
White Male			1 [Reference]	
18 to 44			1.42 [0.96 to 2.11]	0.08
45 to 54			1.43 [1.05 to 1.93]	0.02
55 to 64			1.25 [0.98 to 1.61]	0.07
65 to 74			1 [Reference]	
75 or older			0.44 [0.32 to 0.59]	<0.001
Accessing the internet at home				
Straight	1 [Reference]		1 [Reference]	
Lesbian, gay, bisexual, additional orientations, not sure	1.08 [0.70 to 1.66]	0.73	1.17 [0.76 to 1.82]	0.48
Black Female			1.24 [0.89 to 1.71]	0.20
Hispanic Female			1.70 [1.09 to 2.65]	0.02
White Female			0.67 [0.46 to 0.98]	0.04
Black Male			1.54 [1.20 to 1.99]	<0.001
Hispanic Male			1.50 [1.07 to 2.09]	0.02
White Male			1 [Reference]	
18 to 44			0.68 [0.40 to 1.15]	0.15
45 to 54			0.53 [0.35 to 0.80]	0.003
55 to 64			1.33 [1.02 to 1.74]	0.04
65 to 74			1 [Reference]	
75 or older			0.81 [0.62 to 1.05]	0.11
Assistance with legal issues				
Straight	1 [Reference]		1 [Reference]	
Lesbian, gay, bisexual, additional orientations, not sure	1.43 [0.92 to 2.23]	0.11	1.24 [0.74 to 2.08]	0.42
Black Female			2.01 [1.37 to 2.96]	<0.001
Hispanic Female			1.61 [0.95 to 2.72]	0.08

© 2025 Lamba S et al. *JAMA Network Open*.

	Unadjusted		Adjusted[a]	
	Prevalence ratio [95% CI]	P-value	Prevalence ratio [95% CI]	P-value
Assistance with legal issues (continued)				
White Female			1.10 [0.70 to 1.73]	0.68
Black Male			2.34 [1.68 to 3.27]	<0.001
Hispanic Male			1.71 [1.05 to 2.79]	0.03
White Male			1 [Reference]	
18 to 44			1.32 [0.78 to 2.24]	0.30
45 to 54			1.40 [0.97 to 2.00]	0.07
55 to 64			1.39 [1.05 to 1.85]	0.02
65 to 74			1 [Reference]	
75 or older			0.84 [0.64 to 1.10]	0.21
Transportation for basic needs				
Straight	1 [Reference]		1 [Reference]	
Lesbian, gay, bisexual, additional orientations, not sure	1.39 [0.89 to 2.17]	0.14	1.12 [0.67 to 1.89]	0.66
Black Female			1.28 [0.83 to 1.97]	0.27
Hispanic Female			1.38 [0.79 to 2.41]	0.26
White Female			1.06 [0.66 to 1.71]	0.80
Black Male			1.83 [1.29 to 2.60]	<0.001
Hispanic Male			1.26 [0.80 to 2.00]	0.32
White Male			1 [Reference]	
18 to 44			1.50 [0.91 to 2.48]	0.11
45 to 54			0.77 [0.49 to 1.21]	0.25
55 to 64			0.97 [0.71 to 1.33]	0.87
65 to 74			1 [Reference]	
75 or older			0.64 [0.47 to 0.87]	0.004
Adult caregiving for self or others				
Straight	1 [Reference]		1 [Reference]	
Lesbian, gay, bisexual, additional orientations, not sure	0.91 [0.58 to 1.44]	0.69	0.90 [0.55 to 1.47]	0.66
Black Female			1.28 [0.86 to 1.91]	0.22
Hispanic Female			1.31 [0.77 to 2.22]	0.32
White Female			1.00 [0.65 to 1.53]	0.99
Black Male			1.57 [1.14 to 2.16]	0.006
Hispanic Male			1.20 [0.80 to 1.80]	0.38
White Male			1 [Reference]	
18 to 44			1.03 [0.54 to 1.96]	0.94
45 to 54			0.87 [0.55 to 1.36]	0.54
55 to 64			1.12 [0.81 to 1.54]	0.49
65 to 74			1 [Reference]	
75 or older			1.06 [0.79 to 1.42]	0.69

© 2025 Lamba S et al. *JAMA Network Open*.

	Unadjusted		Adjusted[a]	
	Prevalence ratio [95% CI]	P-value	Prevalence ratio [95% CI]	P-value
Managing experiences of discrimination				
Straight	1 [Reference]		1 [Reference]	
Lesbian, gay, bisexual, additional orientations, not sure	2.31 [1.54 to 3.48]	<0.001	1.79 [1.21 to 2.64]	0.004
Black Female			2.51 [1.69 to 3.74]	<0.001
Hispanic Female			2.41 [1.47 to 3.95]	<0.001
White Female			1.49 [0.94 to 2.35]	0.09
Black Male			2.77 [1.91 to 4.01]	<0.001
Hispanic Male			1.46 [0.91 to 2.34]	0.12
White Male			1 [Reference]	
18 to 44			1.77 [1.16 to 2.69]	0.008
45 to 54			2.07 [1.45 to 2.94]	<0.001
55 to 64			1.77 [1.31 to 2.38]	<0.001
65 to 74			1 [Reference]	
75 or older			0.53 [0.36 to 0.76]	<0.001
Getting additional orientations education or job training				
Straight	1 [Reference]		1 [Reference]	
Lesbian, gay, bisexual, additional orientations, not sure	1.48 [0.94 to 2.34]	0.09	0.79 [0.43 to 1.47]	0.46
Black Female			1.42 [0.90 to 2.23]	0.13
Hispanic Female			1.30 [0.77 to 2.19]	0.33
White Female			0.99 [0.58 to 1.68]	0.97
Black Male			2.09 [1.35 to 3.22]	<0.001
Hispanic Male			1.48 [0.86 to 2.56]	0.16
White Male			1 [Reference]	
18 to 44			6.66 [4.33 to 10.24]	<0.001
45 to 54			3.68 [2.44 to 5.54]	<0.001
55 to 64			2.57 [1.74 to 3.81]	<0.001
65 to 74			1 [Reference]	
75 or older			0.29 [0.16 to 0.53]	<0.001
Getting or maintaining housing				
Straight	1 [Reference]		1 [Reference]	
Lesbian, gay, bisexual, additional orientations, not sure	2.41 [1.40 to 4.15]	0.002	1.91 [1.10 to 3.34]	0.02
Black Female			1.19 [0.79 t 1.79]	0.41
Hispanic Female			1.46 [0.83 to 2.55]	0.19
White Female			0.79 [0.47 to 1.31]	0.36
Black Male			2.08 [1.43 to 3.03]	<0.001
Hispanic Male			1.80 [1.13 to 2.85]	0.01
White Male			1 [Reference]	
18 to 44			1.71 [1.10 to 2.67]	0.02

© 2025 Lamba S et al. *JAMA Network Open*.

	Unadjusted		Adjusted[a]	
	**Prevalence ratio [95% CI]**	**P-value**	**Prevalence ratio [95% CI]**	**P-value**
Getting or maintaining housing (continued)				
45 to 54			1.37 [0.89 to 2.11]	0.15
55 to 64			1.36 [0.98 to 1.90]	0.07
65 to 74			1 [Reference]	
75 or older			0.42 [0.28 to 0.62]	<0.001
Finding or keeping work				
Straight	1 [Reference]		1 [Reference]	
Lesbian, gay, bisexual, additional orientations, not sure	1.71 [1.07 to 2.75]	0.03	1.00 [0.56 to 1.78]	1.00
Black Female			1.19 [0.75 to 1.88]	0.47
Hispanic Female			1.20 [0.69 to 2.08]	0.51
White Female			1.05 [0.63 to 1.76]	0.84
Black Male			1.30 [0.81 to 2.11]	0.28
Hispanic Male			1.15 [0.62 to 2.12]	0.66
White Male			1 [Reference]	
18 to 44			6.16 [3.61 to 10.53]	<0.001
45 to 54			3.12 [1.92 to 5.08]	<0.001
55 to 64			3.07 [1.95 to 4.84]	<0.001
65 to 74			1 [Reference]	
75 or older			0.18 [0.09 to 0.36]	<0.001
Obtaining childcare				
Straight	1 [Reference]		1 [Reference]	
Lesbian, gay, bisexual, additional orientations, not sure	1.67 [0.73 to 3.83]	0.23	0.73 [0.28 to 1.89]	0.52
Black Female			2.41 [0.98 to 5.95]	0.06
Hispanic Female			2.83 [1.17 to 6.84]	0.02
White Female			3.51 [1.36 to 9.07]	0.009
Black Male			1.60 [0.62 to 4.09]	0.33
Hispanic Male			2.21 [0.88 to 5.55]	0.09
White Male			1 [Reference]	
18 to 44			4.14 [1.80 to 9.52]	<0.001
45 to 54			2.03 [0.67 to 6.21]	0.21
55 to 64			1.35 [0.51 to 3.58]	0.54
65 to 74			1 [Reference]	
75 or older			0.34 [0.12 to 1.01]	0.05

Abbreviations: CI, Confidence Interval

[a] Models were adjusted for age-groups and race-ethnicity-sex

© 2025 Lamba S et al. *JAMA Network Open*.
