# Supplementary material for: Health-Related Social Needs Among LGB+ Veterans
Source: JAMA Netw Open. 2025 Oct 29;8(10):e2539986. doi: 10.1001/jamanetworkopen.2025.39986 (PMC12573039; doi:10.1001/jamanetworkopen.2025.39986)
Supplement: Supplement 2. — Data Sharing Statement [file jamanetwopen-e2539986-s002.pdf]

Data Sharing Statement

Lamba. Health-Related Social Needs Among LGB+ Veterans. *JAMA Netw Open*. Published October 29, 2025. doi:10.1001/jamanetworkopen.2025.39986

Data
Data available: No

Additional Information
Explanation for why data not available: We will not make the data available due to VHA security restrictions.
